# The heterogeneity of mesenchymal stem cells: an important issue to be addressed in cell therapy

**DOI:** 10.1186/s13287-023-03587-y

**Published:** 2023-12-20

**Authors:** Jingxuan Li, Zewen Wu, Li Zhao, Yang Liu, Yazhen Su, Xueyan Gong, Fancheng Liu, Liyun Zhang

**Affiliations:** 1 Third Hospital of Shanxi Medical University, Shanxi Bethune Hospital, Shanxi Academy of Medical Sciences, Tongji Shanxi Hospital, Taiyuan, 030032 China; 2 School of Pharmacy, Shanxi Medical University, Taiyuan, 030600 China

**Keywords:** Mesenchymal stem cells, Heterogeneity, Consistency

## Abstract

With the continuous improvement of human technology, the medical field has gradually moved from molecular therapy to cellular therapy. As a safe and effective therapeutic tool, cell therapy has successfully created a research boom in the modern medical field. Mesenchymal stem cells (MSCs) are derived from early mesoderm and have high self-renewal and multidirectional differentiation ability, and have become one of the important cores of cell therapy research by virtue of their immunomodulatory and tissue repair capabilities. In recent years, the application of MSCs in various diseases has received widespread attention, but there are still various problems in the treatment of MSCs, among which the heterogeneity of MSCs may be one of the causes of the problem. In this paper, we review the correlation of MSCs heterogeneity to provide a basis for further reduction of MSCs heterogeneity and standardization of MSCs and hope to provide a reference for cell therapy.

## Introduction

**Fig. 1:**
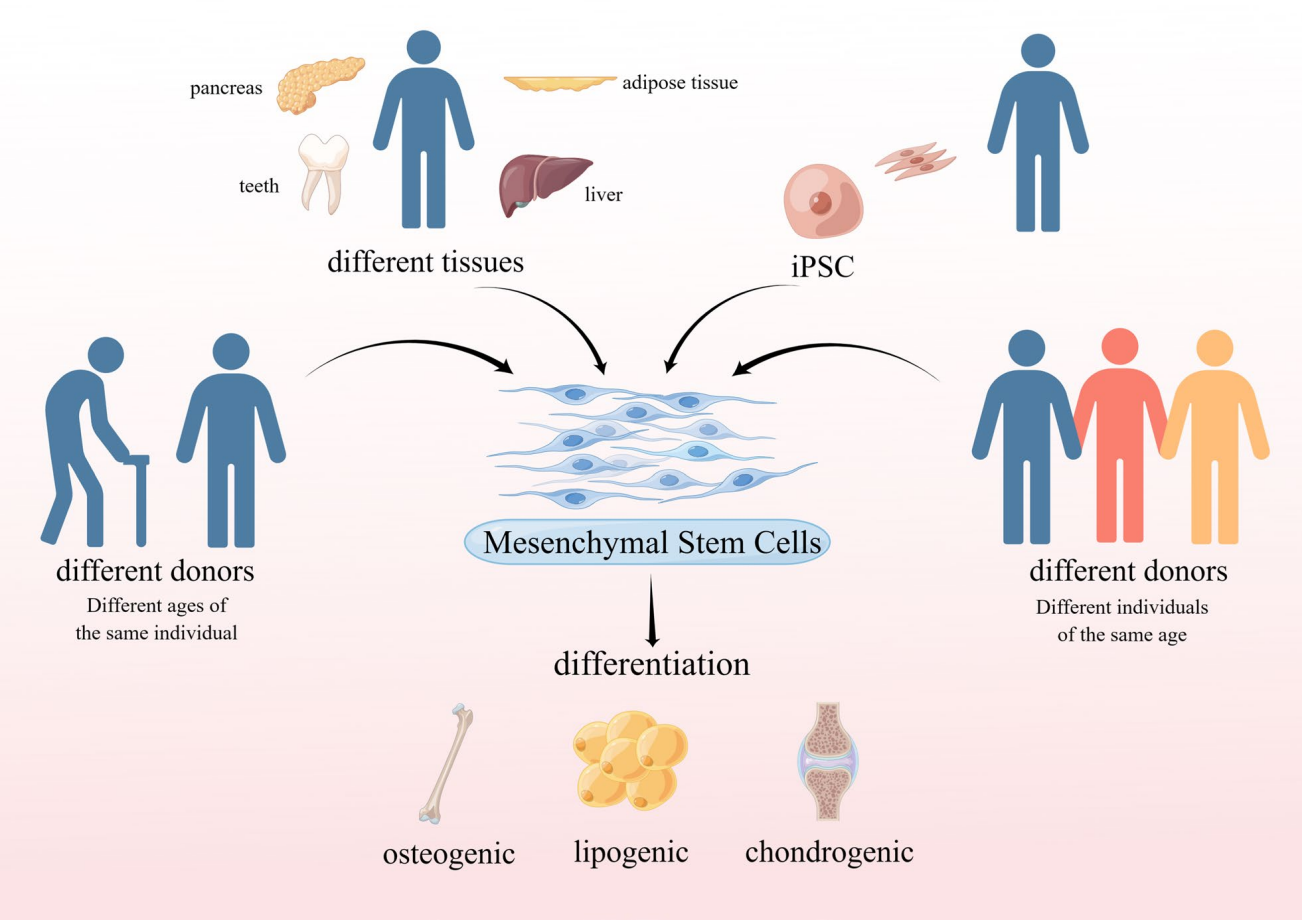
Different source of MSCs

Mesenchymal stem cells (MSCs) are derived from the early mesoderm with high potential for self-renewal and multidirectional differentiation. Since 1968, when Professor Friedenstein first discovered the presence of MSCs in bone marrow, and also isolated and cultured MSCs in vitro using the apposition method [1, 2]. Subsequently, it has been shown that MSCs can be obtained from bone marrow, skin, adipose, umbilical cord and other tissues and organs [3, 4] and have the ability to differentiate into adipocytes, osteoblasts, and chondrocytes [5], and later confirmed the ability of MSCs to self-renew in vivo. Numerous studies have shown that MSCs have at least two important functions: immunosuppression and tissue repair, and paracrine action of MSCs can produce large amounts of cytokines, chemokines, and growth factors to promote tissue injury repair, which is usually considered as the main mechanism for MSCs to participate in tissue injury repair [6]. In 2008, it was suggested in *Nat Rev Immunol* that MSCs act in a "Touch and Go" manner, i.e., by rapidly migrating to the damaged organ and releasing stress-induced therapeutic molecules that are then cleared by the body [7]. MSCs have been widely used in preclinical and clinical studies and have shown satisfactory results in the treatment of various hematologic, cardiovascular, neurological, and autoimmune diseases. For example, MSCs have the ability to improve the function of the patient's islets or transplanted islets, to repair diabetic neuropathy in streptozotocin-induced insulin-deficient T1D mice, and also to repair diabetic neuropathy in high-fat diet-induced T2D mice [8]; MSCs can also enhance chondrogenesis by improving chondrogenesis [9], promoting cartilage regeneration and preventing degeneration [10, 11]. They can also be used in the treatment of skin defects and wound healing. However, while MSCs are receiving more and more attention from researchers and clinical filings are increasing, there are also problems that need to be solved in their use for clinical treatment. Although the safety of MSCs has been verified, the safety of MSCs preparations, such as the problem of microbial contamination, quality control in the transportation of MSCs; on the other hand, the problem of clinical treatment modalities, such as the route of administration, dose and timing of treatment, have not yet formed a clear consensus and standard. With the standardization of quality control, the introduction of international treatment consensus, and the emergence of treatment guidelines for different diseases, it is believed that we can minimize the objective differences. However, more variability is caused by the heterogeneity of MSCs, which is mainly reflected in the following aspects: (1) uncertainty in nomenclature; (2) differences in MSCs from different donors; (3) differences in MSCs from different tissues; and (4) intercellular differences (Fig. 1). Therefore, it is important to solve the problem of heterogeneity of MSCs to enhance their clinical therapeutic effects, as well as productization. In this review, we summarize the current studies on the definition of MSCs and the differences between different sources by summarizing MSCs-related studies, with particular attention to MSCs heterogeneity-related studies, and summarize the results and perspectives in these studies to provide reference for further research and future clinical translation of MSCs.

### Clinical application of MSCs

Since the first stem cell drug indicated for knee cartilage injury repair was approved in 2009, MSCs have shown significant potential in clinical applications. Up to now (November 2023), there are more than 13,300 MSC-related clinical studies in the registry (http://www.clinicaltrials.gov/) using "MSC" as the search term for a drug name. Taking adipose tissue-derived MSCs (AD-MSCs) as one of the most accessible and important sources of MSCs, they are currently primarily used in the following areas: (1) Plastic and esthetic surgery: AD-MSCs are widely utilized in plastic and esthetic surgeries. They can be used for tissue filling and repair, such as treating facial wrinkles, skin scars [12–14], and breast reconstruction [15–17]. (2) Arthritis and cartilage injury treatment: AD-MSCs have demonstrated potential effects in the treatment of arthritis and cartilage injuries. They can promote cartilage tissue regeneration and repair, reduce inflammation, and improve joint function [18]. (3) Wound healing: AD-MSCs possess the ability to promote wound healing and regeneration. They can facilitate neovascularization at the site of injury, stimulate tissue regeneration, and alleviate inflammation. AD-MSCs can be applied directly to wounds or used in combination with techniques such as fat transplantation, hyaluronic acid, biomaterials, and platelet-rich plasma (PRP) to enhance wound healing speed and quality [13, 19, 20]. (4) Treatment of autoimmune diseases: AD-MSCs exhibit immunomodulatory and immunosuppressive effects, making them suitable for treating autoimmune diseases such as rheumatoid arthritis and systemic lupus erythematosus [21, 22]. They can regulate immune responses, alleviate inflammation, and promote tissue repair. (5) Cardiovascular disease treatment: AD-MSCs show potential in the treatment of cardiovascular diseases [23]. They can promote myocardial tissue repair and regeneration, improve cardiac function, and have been used for conditions like myocardial infarction and heart failure. Furthermore, recent research has also indicated potential applications of AD-MSCs and other MSCs in various other fields, including liver disease, lung disease, and kidney disease. In response to the COVID-19 pandemic, MSCs have also demonstrated promising therapeutic effects [24–27], highlighting their strong potential in the field. It is important to note that these are just a few examples of MSCs in the clinic and that specific clinical applications are still in the research and development phase and may be variable depending on the region and medical institution. We will focus on the heterogeneity of MSCs in the following.

## Divergence in the definition of MSCs

There are many different nomenclatures for MSCs, such as "mesenchymal stem cell," "mesenchymal stromal cell," and "multipotent stromal cell." The differences in nomenclature indicate that the current study of the MSCs cell population is still relatively ambiguous. The term mesenchymal stem cell has been widely used since it was officially named 30 years ago [28], mainly to represent a class of cells derived from human and mammalian bone marrow and periosteum that can be isolated from the body and have the ability to expand and induce the formation of a variety of mesenchymal cells and tissues in vitro. Mesenchymal stromal cells are a heterogeneous group of cells that are multipotent with a variety of biological properties, including directional migration, paracrine secretion, immunosuppression, and anti-inflammation effects, and is considered an ideal candidate cell type for repairing tissue damage due to multiple etiologies [29]. The nomenclature of multipotent stromal cells focuses on a limited capacity for self-renewal, but this cell population still has the ability to differentiate along a multi-mesenchymal lineage [30], which does not mean that this cell population is completely free of heterogeneity, as studies have confirmed that there are multiple cell types that share this ability to differentiate along a multi-mesenchymal lineage, with specific phenotypic and functional characteristics in different tissues and different organs. Due to the lack of specificity and unique markers in MSCs cultured in vitro, it is widely accepted that MSCs must express CD105, CD73, and CD90 and lack CD45, CD34, CD14 or CD11b, CD79a or CD19, and HLA-DR surface molecules expressed [31]. The core reason for the discrepancy in nomenclature is that the vast majority of MSCs are pericytes derived from the perivascular (vessel wall) [32], whereas it has been demonstrated through several in vivo and in vitro studies that pericytes isolated from various tissues can produce MSCs and have immunomodulatory and trophic functions [33]. Different definitions encompass different combinations of specific cells, so it is essential to use a clear definition. However, the lack of a precise definition of the constituent cell types makes it impossible to predict the overall behavior of these heterogeneous populations. Thus, it also leads to variability in clinical studies. With the advancement of research tools, such as further development of technologies like single-cell sequencing, the implementation of new meticulous international identification standards will help the promotion and standardization of clinical studies of MSCs.

## Inter-individual variation

MSCs have been identified in an internationally accepted manner and criteria, and in 2006 the International Society for Cell & Gene Therapy (ISCT) established the basic criteria for the definition of MSCs [31], which are also the minimum criteria for the identification of MSCs: (1) an appressed growth state under standard in vitro culture conditions; (2) greater than or equal to 95% of cells expressing CD105, CD73, and CD90, and no more than 2% of the total number of cells expressing CD45, CD34, CD14, CD11b, CD79a, CD19, or HLA-II class molecules; (3) the ability to differentiate into osteoblasts, chondrocytes, and adipocytes under in vitro induction conditions. However, the biological differences between MSCs from different individuals cannot be ignored. The differences between individuals are mainly reflected in two aspects: On the one hand, longitudinal comparisons show differences between MSCs of different ages; on the other hand, horizontal comparisons show differences between MSCs of different individuals of the same age, for example, MSCs from adult and neonatal skin show significant differences in lipogenesis and chondrogenesis [34]. Differences also existed between MSCs of the same tissue origin in the elderly and infants. Aging is a universal feature of all organisms and is considered a high-risk factor for many diseases from a medical perspective. There is evidence that MSCs also undergo functional decline during organism development as the organism ages [35–37]. The aging of MSCs with age is manifested by enlargement, telomere shortening or p53/p21-mediated accumulation of DNA damage, impaired DNA methylation or histone acetylation, and elevated levels of reactive oxygen species (ROS) and nitric oxide (NO) [38]. In humans and mice, once born, the content of MSCs in the bone marrow decreases continuously, and aging of the organism reduces the density of MSCs and their osteogenic potential [39, 40]. In addition, there is growing evidence that MSCs aging favors adipogenesis at the expense of osteogenesis, leading to impaired bone formation [41–43]. This is consistent with the in vivo situation, where adipose tissue in the bone marrow increases with aging. This situation may be related to the reduced expression of activator proteins of the PDZ structural domain thereby enhancing the expression of peroxisome proliferator-activated receptor γ (PPAR-γ), which is associated with adipogenesis and suppresses the expression of Runt-related transcription factor 2 (RUNX2), which is associated with osteogenesis [44]. Furthermore, in a study on MSCs of adipose origin, adult MSCs (individuals > 40 years of age) produced significantly higher levels of IL-6 and IL-8 than MSCs from individuals younger than 16 years of age [45], and this enhanced pro-inflammatory secretome may significantly reduce the immunomodulatory capacity of MSCs. Although the mechanisms of inflammation production are unknown, it has been proposed that adipose tissue is a major producer of systemic pro-inflammatory cytokines with age [46–48]. In particular, through senescence-associated secretory phenotype (SASP), aging cells themselves produce many pro-inflammatory cytokines and chemokines [49, 50]. The accumulation of adipose tissue and adipocytes was observed in the bone marrow of the elderly [51] and mice [52]. Notably, the increased number of adipocytes may be due to the bias of aging MSCs toward lipogenic differentiation [53]. Since senescence represents a decline in systemic function, all cells in the bone marrow should be subjected to similar senescence stress, which affects neighboring cells. In addition, senescence reduces the expression level of Fibroblast Growth Factor-2 (*FGF-2*) in most cells, which may lead to a decrease in the proliferative capacity of MSCs [54]. According to studies with aging, the Wnt/β-catenin signaling pathway is increased in aged mice, but its function needs to be further investigated [55, 56]. Although MSCs offer possibilities for the treatment of various diseases, aging and aging-related processes can significantly affect the outcome of stem cell therapy. Baster et al. [57] showed that the number of bone marrow-derived MSCs (BM-MSCs) with osteogenic potential decreases during early human aging, which may be associated with age-related reduction in bone formation, mechanical properties, and integrity of bone. In addition to MSCs, neural stem cells (NSCs), satellite cells, and hematopoietic stem cells (HSCs) have also been reported to exhibit age-related decreases in proliferation and differentiation potential both in vitro and in vivo [58, 59]. The loss of some functional stem cells with age can have profound effects on tissue viability. The mechanisms of stem cell depletion are unclear, but are most likely due to a combination of many intrinsic and extrinsic factors, including changes in growth factor activity, accumulation of DNA damage, and decreased progenitor cell responsiveness. Therefore, it is essential to consider the age of the donor tissue and recipient environment in any treatment based on MSCs transplantation. There are also differences in MSCs from different donor sources. A study published in *Stem Cell Res Ther* [60], 13 hMSC samples from 10 "healthy" donors were assessed for donor variability and tissue origin differences in single-cell gene expression profiles, indicating that there is a donor effect on the expression of a gene in MSCs and that the donor effect on MSCs is mainly on the cell cycle. Subtle differences were found between the BM-MSCs and cord tissue-derived MSCs (CT-MSCs) groups in cytokines (IL-1β, IL-8, IL-12, and IL-17), anti-inflammatory cytokines (IL-1RA, IL-13), chemokines (MCP-1, MIG, MIP-1α, MIP-1β, and RANTES), and pro-angiogenic (VEGF) markers. Researchers observed differences in growth factors (HGF and G-CSF) between BM-MSCs and CT-MSCs. We found significant donor differences in the UCT and BM-MSCs groups, suggesting that the cell cycle is indeed a major driver of MSCs heterogeneity. In summary, differences exist both in different ages of the same individual and in different individuals of the same age (Table 1).

**Table 1:** Effects of cellular aging on MSCs

	Aged MSCs	References
Morphology	increase in cell size; contain more actin stress fibers; telomere attrition	[61, 62]
Differentiation	loose osteogenic potential; gain adipogenic potential	[42, 43, 53]
Growth rate	decrease	[63]
Pathway	more positive for SA-βGAL activity; the upregulation of the p53 pathway	[64]

## Heterogeneity of different tissue sources

Currently, MSCs can be isolated from many different tissues, including adipose tissue 65], skin tissue [66], blood [67], umbilical cord blood [68], teeth [69], pancreas [70], and liver [71]. Cells from different tissues are capable of tri-lineage differentiation (osteogenic, chondrogenic, lipogenic) and display similar surface markers [34, 72], but still differ significantly in content, proliferative capacity, immunomodulation, and differentiation capacity. (1) There are differences in the content of MSCs from different tissue sources. According to the presenting as cell clone-forming units (CFU-F) assay, the content of MSCs of bone marrow source is roughly 0.001–0.01% of mononuclear cells (MNCs) [73, 74], while placental amniotic membrane and umbilical cord-derived MSCs (UC-MSCs) account for 0.2–1.8% of MNCs [75]. The extremely low number of MSCs in umbilical cord blood results in a probability of isolating and successfully culturing MSCs from cord blood of only 5.7–10% [76]. It is not clear what the amount of MSCs from individual adipocytes is, but it has been reported that it does not exceed 50% [77]. (2) There are differences in the proliferative capacity of MSCs from different tissue sources. It has been shown that human umbilical cord perivascular cells (HUCPVCs) show a higher proliferative potential than BM-MSCs and are able to differentiate into bone, cartilage, and adipose, and in addition, HUCPVCs exhibit higher levels of CD146 relative to BM-MSCs [78]. Since the genetic background of the mouse models is highly consistent, the comparative data of MSCs from different tissues of mice are more meaningful and convincing. Compared to BM-MSCs, AD-MSCs have higher proliferative activity and produce more vascular endothelial growth factor (VEGF) and hepatocyte growth factor (HGF) [79–81]. MSCs are age-specific, with a marked decrease in the number and proliferative capacity of BM-MSCs with increasing age. Interestingly, gender also affects the proliferative capacity of MSCs, with female BM-MSCs having a cell diameter of 20.9 ± 0.8 µm and a doubling time of approximately 3.3 ± 1.9 days, while male BM-MSCs have a cell diameter of 22.0 ± 1.1 µm and a doubling time of approximately 5.0 ± 3.7 days [82]. (3) MSCs from different tissue sources also differ greatly in their immunomodulatory and differentiation capacity. Data from genetic aspects surface that UC-MSCs and amniotic membrane origin have a higher immunomodulatory potential, while MSCs of bone marrow origin have a higher potential to support regenerative development, such as neuronal development and differentiation [83, 84]. The experimental results showed that AD-MSCs have a stronger adipogenic differentiation capacity and produce more cellular matrix components, which may be due to the high expression of fatty acid-binding protein FABP4 in AD-MSCs compared to BM-MSCs [85]. In terms of gene expression, MSCs at the umbilical cord junction showed the least difference from UC-MSCs, whereas AD-MSCs differed significantly from MSCs of other sources in terms of protein expression, most likely due to the longer developmental time and higher differentiation of AD-MSCs compared to MSCs of other sources. A comparative study of human and mouse-derived MSCs found that, compared with hMSCs, Muse-MSCs exhibited higher expression levels of the *p53* repressor MDM2; signal acceptance-related genes *EGF*, *VEGF*, *PDGF*, *WNT*, *TGFB*, *INHB*, and *CSF*; ribosomal protein; and glycolysis and oxidative phosphorylation. Conversely, hMSCs had higher expression levels of *FGF* and *ANGPT*; *Rho* family and caveola-related genes; amino acid and cofactor metabolism; MHC class I/II, and lysosomal enzyme genes than Muse-MSCs [86]. Currently, MSCs are commonly found in three major tissue sources: bone marrow, umbilical cord, and adipose. In summary, in terms of cell abundance, umbilical cord is the most abundant, followed by bone marrow and adipose sources, and the least abundant in umbilical cord blood; for the proliferative capacity of MSCs, due to their age-specific characteristics, UC-MSCs have a clear advantage, followed by adipose and bone marrow; in terms of immunomodulation, MSCs from umbilical cord, amniotic membrane, and adipose sources are superior to MSCs from bone marrow, and MSCs from placental sources have the least immunomodulatory capacity (Table 2).

**Table 2:** Comparison of the sources of MSCs and their characteristics

Source	Advantages	Disadvantages	Multiplication capacity	Markers	Clinical applications	References
BM	Potential to differentiate into hepatocytes Expression of cytochrome P450 Multiple clinical trials have demonstrated its safety and efficacy	The acquisition process is often painful and comes with the risk of infection Cell yield and differentiation potential depend on donor characteristics (e.g. age)	Show a slow proliferation rate, DT time usually takes 40–60 h, and signs of senescence are already present even by the 6th/7th generation of the passages	In addition to surface markers (CD90, CD105, CD73) MSCs also express cell adhesion molecules such as CD29, CD44 and CD105, while CD14, CD34 and CD45 are not expressed	Treatment of orthopedic diseases characterized by extensive bone damage BM-MSCs can also be used to treat non-arthritic, femoral head necrosis Potential therapeutic effect on myocardial infarction as well as GVHD and SLE	[87–89]
AD	Adipose tissue is abundant and readily available and secretes a number of angiogenic and apoptotic cytokines AD-MSCs are more immunosuppressive than BM-MSCs	AD-MSCs have poorer osteogenic and chondrogenic potential compared to BM-MSCs	Better performance than BN-MSCs, with DT shortened to 20–45 h and no signs of senescence at the time of passing to the eighth generation	High expression of surface markers (CD90, CD105, CD73) and low expression of hematopoietic markers (CD45, CD34) in MSCs	Immunosuppressive GVHD treatment Application to cosmetic or dermatological conditions Successfully used to treat skeletal muscle injuries, meniscal injuries and tendon and peripheral nerve regeneration	[87, 88, 90, 91]
UC	High ubiquity, avoiding invasive examinations and ethical issues Exhibits higher expansion and migration capacity than BM-MSCs	UCB-MSCs may not have lipogenic differentiation potential May be inferior to BM or blood in terms of osteogenic differentiation potential	WJ-MSCs, for example, show the highest value-added rate, 3–4 times higher than AT-MSCs and BM-MSCs	Most of the immunomarkers were expressed similarly to BM-MSC, except that the expression of HLA-ABC and CD106 was lower in UC-MSC than in BM-MSC	In vitro regeneration of pancreatic islet cells Treatment of GVHD and SLE	[87, 92, 93]

## Heterogeneity of MSCs of the same tissue origin

It is generally assumed that cell clusters derived from single-cell clones should be homogeneous, but this is not really the case. Clonal cell masses grown in single-cell clone cultures of BM-MSCs usually do not accurately represent either a homogeneous stem cell population or a specific function of stem cells derived from a single cell [94]. Just as there are no identical leaves in the world, there are no identical cells. It was found that the clonal cell clusters (non-single-cell clones) that emerged from BM-MSCs in apposed culture were only about 35% derived from single cells [94]. BM-MSCs are MSCs with pluripotent properties and are considered as a potential approach for the treatment of several diseases. Since 1995, several clinical trials have progressed, but to date MSC therapy has not been applied on a large scale. One factor contributing to the unsuccessful clinical trials is the heterogeneity among individual participants, which can be attributed to the heterogeneity among the patients treated as well as the heterogeneity among bone marrow donors. However, even cells from a single donor can show functional heterogeneity (including proliferation rate, number of colonies formed, differentiation potential) under the same culture conditions. However, heterogeneity in stem cell culture processes is often overlooked and underestimated in many studies. It has been demonstrated that colonies of BM-MSCs from the same donor (single biological cell source) differ significantly in terms of colony size, fusion, and multidirectional differentiation potential [95, 96]. It has been demonstrated that few individual cells are formed under sparse inoculation conditions (30 cells/cm^2^). Regardless of the source, most cells showed heterogeneity in proliferative capacity and biological properties after only four days of growth, and even the most homogeneous colonies of single-cell origin are affected by neighboring progeny and exhibited heterogeneity within seven days, suggesting that the appearance of heterogeneity is inevitable even in specific stem cell colonies. According to the analysis, physical contact between cells alone does not change the biophysical properties or the ability of cells to add value [94]. In 2017, Professor Davies from the University of Toronto, Canada, suggested that "umbilical cord MSCs share the same population heterogeneity as BM-MSCs and that UC-MSCs gradually lose several differentiation functions and homogenize into 'fibroblasts' as culture time increases" [97]. However, in reality, UC-MSCs still have the ability to differentiate toward lipoblasts and osteoblasts after long-term culture [98]. When two different populations of isogenic MSCs(UCB1 and UCB2) were isolated from cord blood for comparison, UCB1 exhibited faster growth kinetics, higher population multiplication capacity, and higher lipogenic capacity compared to UCB2, and the UCB2 population had a higher osteogenic differentiation capacity; moreover, the gene expression profiles were not consistent, with only 121 genes co-expressed [99, 100]. Heterogeneity was also present in AD-MSCs, starting with the fact that the content of MSCs in adipose tissue was not constant, and there were no differences in the number, proliferation, and differentiation potential acquired by adipose MSCs isolated from the abdominal and gluteal regions. After several generations of adipose isolated MSCs in adipose culture, the expression of CD34 and HLA-DR decreased to less than 2% or was negative [101]. However, CD34 expression can persist for 10–20 weeks if AD-MSCs are cultured with M199 medium supplemented with factor aFGF. Cloning experiments in 96-well plates of BM-MSCs revealed that 50% had the ability to differentiate in three directions, 14% only in two directions, while 1% of the cloned cells had the ability to differentiate in one direction [102]. Single-cell cloning experiments of AD-MSCs also showed that not all AD-MSCs had the ability to differentiate tridirectionally, with approximately 81% having the ability to differentiate unidirectionally and 52% having the ability to differentiate bidirectionally or tridirectionally [95, 103]. BM-MSCs are similarly heterogeneous, with the CD200 + subpopulation in mouse BM-MSCs having a strong osteogenic capacity, whereas the SSEA4 + subpopulation has a strong lipogenic differentiation capacity but lacks osteogenic capacity, and the expression of the CD140a + subpopulation in lipogenic cells is not associated with osteogenic efficiency. BM-MSCs are currently isolated and identified using a combination of non-specific cell surface markers, such as high level expression of CD271, CD44, CD105, CD73, and CD90 and low level expression/non-expression of CD45, CD34, CD14 or CD11b, CD79a or CD19, and HLA-DR. Among these markers, CD271 exhibits extremely high efficiency, but recent studies have found that CD45 and CD34, which have been thought to be inexpressed, are expressed in a small proportion of BM-MSCs [104]. Also only about 50% of MSCs were found to be positive for CD105. So, are these cells MSCs for the minimum criteria established in 2006 for MSCs, i.e., CD73, CD90, CD105 ≥ 95%, CD11b or CD14, CD34, CD45, CD19 or CD79a, and HLA-DA ≤ 2%? MSCs are identical to humans in that the entire genome is not fully conserved, and the presence of genetic polymorphisms will result in differences between the offspring and the parent cells, which may not have an impact on the overall function, but may have an impact on the intensity of certain functions.

## Plasticity of MSCs

**Fig. 2:**
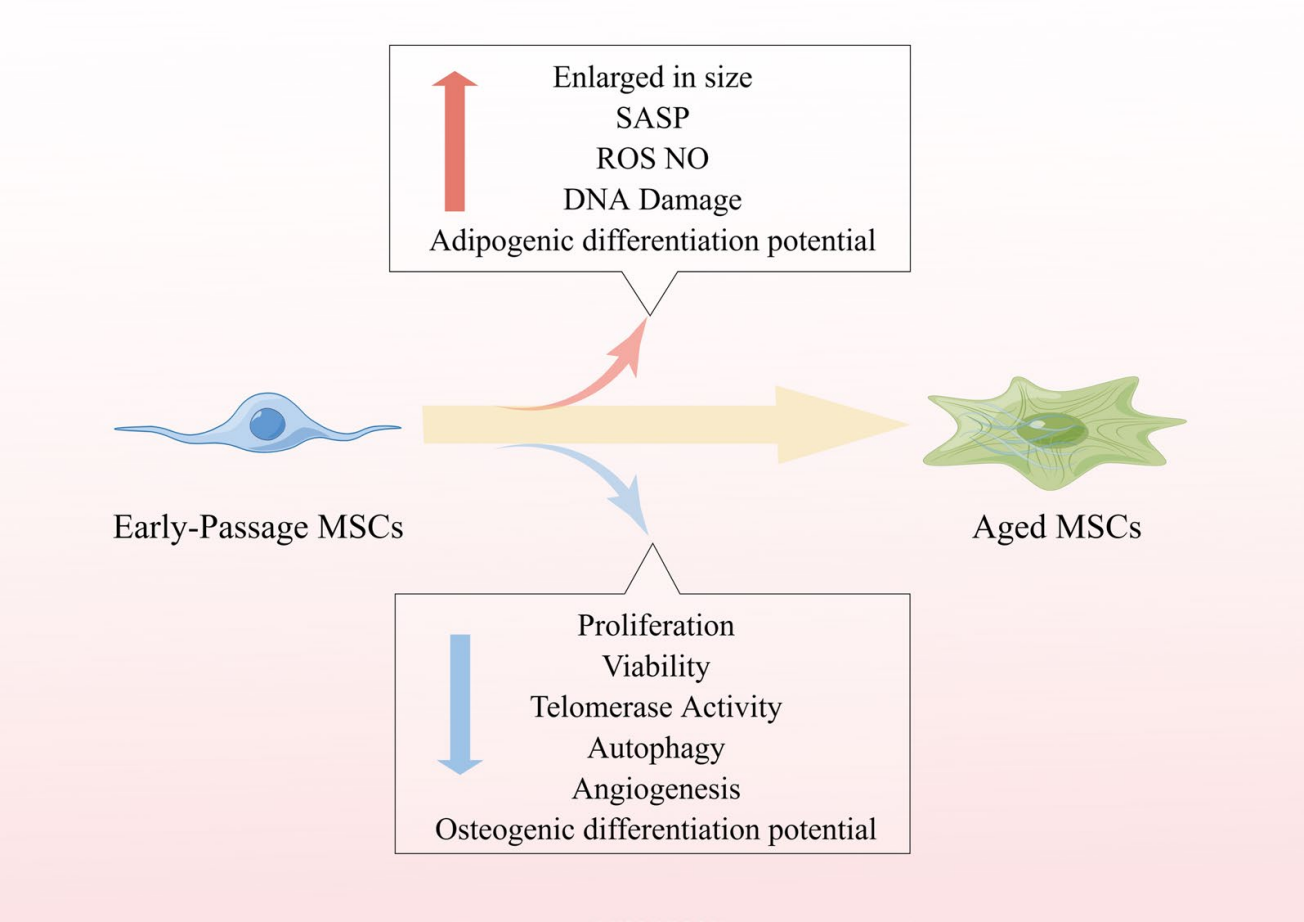
The impact of aging on MSCs

MSCs obtained from different disease states, tissue sources, and donors exhibit variations (Fig. 2). However, even MSCs derived from the same adipose tissue source can have differences in population doubling time and growth rate when obtained from different locations such as the skin, abdomen, and subcutaneous fat [105]. Studies have shown that MSCs sourced from UC-MSCs and human amniotic fluid (hAF-MSCs) possess broader differentiation potentials [106], while placenta-derived MSCs have a lower potential for adipogenesis. Nguyen et al*.* compared BM-MSCs with MSCs derived from the acetabulum and femur and found that bone marrow and femur-derived MSCs formed more calcium deposits during osteogenic differentiation, while BM-MSCs exhibited a stronger role in chondrogenic and adipogenic differentiation processes [107]. Furthermore, MSCs obtained from the femoral head demonstrated a stronger chondrogenic induction capacity compared to MSCs sourced from the iliac crest and vertebral bone marrow [108]. What causes heterogeneity in MSCs of different origins? A very important reason may be the niche of MSCs. In 1978, the concept of MSCs niche was defined as the place where stem cells accumulate in vivo and the environment allows them to remain in an undifferentiated state [109]. Stem cells are regulated by their niches, which leads to the tissue specificity of stem cells [110]. All components of the niche act together to control the cellular and non-cellular components of adult stem cells, and these interactions usually include two mechanisms, namely physical contact and diffusion factors; cell adhesion proteins play a role in intercellular adhesion, differentiation and polarity of MSCs, and are associated with the Wnt pathway involved in the MSCs niche, which will affect their function [111, 112]. The canonical Wnt signaling pathway is involved in the osteogenic and chondrogenic differentiation of MSCs while inhibiting adipogenic differentiation. Additionally, the Wnt/β-catenin pathway is implicated in abnormal skeletal development, including endogenous chondrosarcoma and osteosarcoma [113]. Therefore, the effects occurring during MSC therapy for tumors are closely related to the type of tumor [114]. For instance, a study on acute lymphoblastic leukemia (ALL) demonstrated that the upregulation of specific T-cell receptors (TCRs) in MSCs facilitated the body's anticancer response [115]. Due to the local and systemic interactions of MSCs with other niche cells, the different niche in which MSCs are located may result in different secretion profiles of MSCs, leading to heterogeneity in the different sources of MSCs. A study on interleukins found that IL-1α, IL-1β, and IL-2 induce an immunosuppressive phenotype in MSCs [116–118]. However, there are some problems in explaining the heterogeneity of MSCs using niche alone. A review of recent studies revealed that it is not possible to classify MSCs simply by the presence of tissue sites; a simple example is that osteoblasts that should have developed in bone tissue can also be found in other parts of the body such as heart or muscle during ectopic bone development. Longitudinal studies of MSCs have revealed that these cells are dynamic and can change their function by rapidly altering gene expression. Due to the long-term focus on the therapeutic potential of in vitro expanded MSCs, human knowledge of the phenotype of MSCs has been obtained from in vitro culture, which led to a long period of time where the non-expression of CD34 was used as a criterion for MSCs, but as research progressed it was gradually recognized that CD34 may be expressed in vivo but rapidly downregulated during in vitro culture [119–121]. These have been hindering the understanding of the role of human MSCs in homeostasis and pathology in vivo. On the other hand, the advent of single-cell RNA sequencing (scRNA-seq) has allowed precise identification of corresponding cell subpopulations in humans and mice, and there has been agreement through lineage tracing studies on showing a common surface phenotype of MSCs, but comparative analysis of scRNA-seq datasets has revealed an additional nomenclatural heterogeneity [86], namely that even within the same species, different research groups uniformly refer to overlapping cell populations as MSCs or fibroblasts depending on the focus in their studies, giving the false impression of the existence of many cell populations with different properties [122]. In addition, MSCs are altered accordingly when cultured in vitro. Under in vitro culture conditions, MSCs can be induced to differentiate toward specific cell lines by modulating specific factors and conditions in the culture medium, and this alteration in differentiation potential may also cause phenotypic changes in MSCs [123, 124]. Besides, a number of commonly expressed surface markers, such as CD44, CD73, CD90 and CD105, may also change during the course of in vitro culture. Some surface markers may decrease or disappear when MSCs are cultured in vitro, for example, the markers CD34 and CD45, which are associated with hematopoiesis, gradually decrease during the culture process. Moreover, the surface markers of MSCs may also undergo transformation, such as the expression of CD146, a cell adhesion molecule that is mainly involved in cell–cell or cell–matrix interactions, during the early stages of cell culture. However, as MSCs are further cultured and proliferate, they may gradually lose the expression of CD146. During the course of in vitro culture, MSCs may express some additional surface markers related to proliferation, differentiation or cellular activity. For example, in an inflammatory environment MSCs may express more major histocompatibility complex (MHC) class II, which can affect the function of MSCs in immunomodulatory and anti-inflammatory actions [125, 126]. Changes in in vitro culture can also lead to differences in clinical treatment. Therefore, clarifying the functional and phenotypic status of MSCs and fibroblasts in different niches is the key to solving the MSCs heterogeneity puzzle.

## Discussion and outlook

As of 2023, there have been more than 13,300 MSCs-related clinical studies registered on the ClinicalTrials.gov website, indicating that MSCs have a promising application as a stem cell drug for a number of diseases. For example, MSCs have been approved in the European Union for the treatment of Crohn's complicated by intestinal fistula, and in Korea, Japan, and Canada for the treatment of GVHD [127]. Although MSCs have been approved for marketing in some countries, there is always confusion about the application of MSCs, such as dose differences [128]. In the treatment of RA, we can see that even with the same source of MSCs, the cell volume, and efficacy vary. The most important reason for this is the heterogeneity of MSCs in the treatment of different diseases. In summary, the heterogeneity of MSCs is mainly reflected in the following aspects: (1) Cell proliferation and differentiation capability: MSCs derived from different sources and donors may exhibit variations in their cell proliferation and differentiation potential. This can result in inconsistent growth rates and differentiation levels among different MSCs during the treatment process, thereby affecting the treatment outcome [129]. (2) Cytokine secretion: MSCs regulate tissue repair and immune response through the secretion of cytokines and growth factors. MSCs from different sources and donors may differ in their cytokine secretion profiles. This can lead to variations in the quantity and types of cytokines released by different MSCs during treatment, thus influencing the therapeutic effect [60, 130]. (3) Individual differences and microenvironmental influences: MSCs from different individuals may vary in biological characteristics and functions, which can be influenced by factors such as age, gender, health status, and genetic background. These individual differences and microenvironmental factors can impact the biological behavior and therapeutic effects of MSCs, including cell proliferation rates, differentiation potential, and immunomodulatory abilities [58, 60, 131]. Overall, the heterogeneity of MSCs may lead to variations in their biological behavior and treatment outcomes among different cell populations. This presents a challenge in determining the optimal MSC therapy regimen, dosage, and timing, requiring further research and clinical practice to address these issues. By delving into the heterogeneity and individual differences of MSCs, a better understanding of their impact on treatment outcomes can be achieved, leading to the development of personalized treatment strategies for achieving optimal clinical results. Therefore, it is important to standardize the MSC and thus reduce the effect of heterogeneity. (1) UC-MSCs have the great advantage of large-scale expansion and standardization compared to MSCs of other origins. When UC-MSCs are sorted into surfaces based on their size, smaller cells grow faster and age more slowly than larger cells. In addition using multicolor lentiviral genetic barcode labeling as a clonal developmental analysis revealed that heterogeneity of MSCs can be reduced in their in vitro expansion. (2) It has been shown that pretreatment with inflammatory cytokines (IFNγ and TNFα) can improve the therapeutic effect and after treatment of MSCs showed some consistent changes in gene expression [132–134]. Further cell cycle-based analysis showed that limited heterogeneity was strongly associated with the entry of these cells into the G2/M phase [100, 135], which is also one of the ways to reduce heterogeneity. (3) Induction of MSCs using cells differentiated from iPSCs showed good consistency. Since iPSCs can be taken from multiple sources of somatic cells in vivo and show the ability to grow and proliferate indefinitely, they have an advantage over somatic-derived MSCs. MSCs have been applied in several fields such as tissue engineering and regenerative medicine, which are considered as potential means of treating various inflammatory diseases, especially the discovery of immunomodulatory effects brings light to the treatment of inflammatory diseases. Although optimal criteria for MSCs dose, donor, culture conditions, administration routes, patients, and clinical evaluation criteria have not been established, we believe that further research on MSC heterogeneity and the search for theories and ways to standardize MSCs will be important for the future development of MSC therapy, which still has a bright future and is important for the treatment of various diseases.

## Conclusions

MSCs is considered to be a potential therapeutic approach for various diseases due to its ability of self-renewal and multidirectional differentiation. However, there are great differences between individuals in the application process, and the difference of MSCs is considered to be the biggest factor in the difference of curative effect. There are differences in different individuals from the same species; there are differences in different organizations from the same individual; even from the same batch of MSCs, different colonies have different proliferation and differentiation potential. In order to seek better therapeutic effect, it is very important to obtain more stable, uniform and functional MSCs. Based on the study of iPSC, induced pluripotent stem cell (iPSC)-derived mesenchymal stem cells (iMSCs) have good consistency, and iMSCs are more stable in proliferation, tissue repair, and differentiation applications than other types of tissue-derived MSCs [136]. In addition, in the process of cell therapy, the problem of tumorigenicity that puzzles researchers has also been solved to a certain extent, and iMSCs greatly reduce the possibility of tumor formation. iMSCs have great potential in commercialization and can provide new sustainable and stable products for disease treatment. In conclusion, the most important thing for the treatment of MSCs at present is to solve its heterogeneity, seek a unique and accurate definition of MSCs, reduce the heterogeneity of MSCs to obtain a more inclusive therapeutic effect, and provide more possibilities for cell therapy.

## Data Availability

Please contact the corresponding author for data requests.

## Abbreviations

MSCs: Mesenchymal stem cells
PPAR-γ: Peroxisome proliferator-activated receptor-γ
VEGF: Vascular endothelial growth factor
HGF: Hepatocyte growth factor
BM-MSCs: Bone marrow-derived mesenchymal stem cells/stromal cells
AD-MSCs: Adipose tissue-derived MSCs
CT-MSCs: Cord tissue-derived MSCs
BM: Bone marrow
UC: Umbilical cord
SASP: Senescence-associated secretory phenotype
UCB: Umbilical cord blood
WJ: Wharton’s jelly
PRP: Platelet-rich plasma
DT: Doubling time
GVHD: Graft-versus-host disease
SLE: Systemic lupus erythematosus
MHC: Major histocompatibility complex
iPSCs: Induced pluripotent stem cells
iMSCs: Induced pluripotent stem cell (iPSC)-derived mesenchymal stem cells
